# Exploring the thermal decomposition of cigarette butts and its role in chromium adsorption processes

**DOI:** 10.1016/j.heliyon.2025.e42162

**Published:** 2025-01-23

**Authors:** Carlos Felipe Herrera-Puerta, Santiago Suspes-García, Diana M. Galindres-Jimenez, Diego Cifuentes-Galindres, Luz Elena Tinoco, Juan Carlos Moreno Piraján, Liliana Giraldo Gutierrez, Yesid Murillo-Acevedo

**Affiliations:** a Grupo de Investigación en Ciencias y Educación (ICE), Facultad de Ciencias y Humanidades, Universidad de América, 111711, Bogotá, Colombia; b Grupo de Sólidos Porosos y Calorimetría, Facultad de Ciencias, Universidad de los Andes, Bogotá, 111321, Colombia; c Grupo de Calorimetría, Facultad de Ciencias, Universidad Nacional de Colombia, Bogotá, 111321, Colombia; d Faculty of Health Sciences, Universidad Católica de Ávila, Calle Los Canteros s/n, 05005, Ávila, Spain

**Keywords:** Adsorption, Activated carbon, Thermogravimetry, Cr (VI) adsorption, Cigarette butts

## Abstract

Smoked cigarette butts (SCB) can serve as precursors for producing activated carbon (AC) due to their high carbon content, which originates from cellulose acetate and other components retained during tobacco combustion. This study examines the pyrolysis process and char formation from SCB residue. Kinetic analysis of char formation is conducted using thermogravimetry with heating rates of 5, 10, and 15 °C/min. Mathematical models, including Kissenger-Akahira-Sunose (KAS), Starink (STK), and Ozawa-Flynn-Wall (OFW) are applied to determine thermodynamic parameters, such as activation energy. The resulting char undergoes KOH activation to enhance surface area and pore volume, resulting in BET surface area and micropore volume values of 433 m^2^/g and 0.25 cm^3^/g, respectively. The activated carbon is then used for Cr(VI) removal, exhibiting a maximum adsorption capacity (Q_max_) of 55.8 mg/g, as determined by the Langmuir model.

## Graphical abstract

**Figure:**
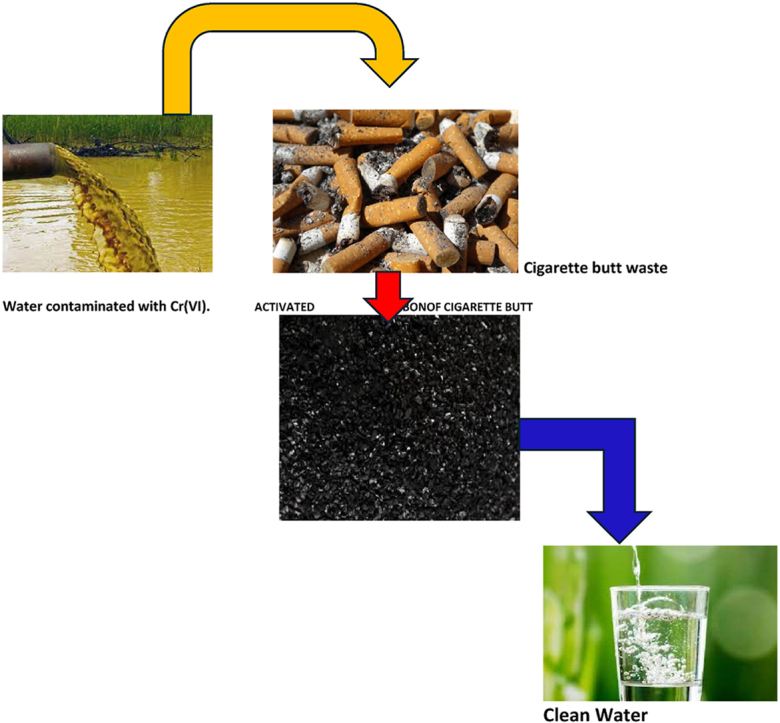

## 1 Introduction

Smoked cigarette butts (SCBs) are a significant form of litter, with the European Union identifying them as one of the top ten debris items found on beaches [1]. The World Health Organization (WHO) estimates that there are 1.3 billion smokers worldwide [2], leading to the annual production of 4.5 trillion cigarette butts [3]. This considerable environmental challenge underscores the urgent need for innovative strategies to manage SCB waste and mitigate its negative impacts on ecosystems and public health.

In Colombia, the National Administrative Department of Statistics (DANE) reports that approximately 16 million Colombians have consumed cigarettes at least once. For instance, in 2016, it was estimated that 94.9 million single-use cigarette butts (SCBs) were generated in Bogotá alone, equivalent to 16 tons of waste found in public spaces such as bars, nightclubs, and other leisure areas [4]. One of the major issues associated with this type of waste is its slow decomposition rate, primarily due to the microbial breakdown of its key components: plasticized cellulose acetate and an outer paper cover [1,5,6]. Despite this, the disposal of SCBs remains a global problem, as they often enter sewage systems, pollute surface water bodies (such as rivers and lakes), and contribute to surface runoff and general waste accumulation.

SCBs can leach into water sources, negatively impacting ecosystems [5]. Over 7,000 chemical compounds have been identified in SCBs, with approximately 1,000 of these compounds present in the tar phase. Notable examples include polycyclic aromatic hydrocarbons (PAHs), nitrosamines, benzene, and heavy metals such as cadmium and nickel, all of which are considered carcinogens [[7], [8], [9], [10]].

To minimize the impact of this type of waste, studies have explored incorporating SCBs into various industrial processes to enhance their value. Examples include the production of fired clay bricks [10], mixing with asphalt [11], cellulose pulp production [12], creating corrosion inhibitors [13], and developing porous materials [14], among other applications [15]. One notable application is the production of carbonaceous materials. SCBs mainly consist of cellulose acetate, a valuable raw material for producing activated carbon (AC).

Activated carbon (AC) is recognized as a universal adsorbent due to its high adsorption capacity and wide-ranging applications across various industries [16]. Its large surface area, porous structure, and unique surface chemistry enable it to adsorb a wide variety of contaminants from air and water [17,18]. The main advantage of AC lies in its ability to tailor solutions for specific adsorption needs [19]. Additionally, its cost-effectiveness, environmental compatibility, and regulatory compliance further enhance its standing as a superior adsorbent [20].

In practice, AC demonstrates its versatility by effectively adsorbing a range of pollutants. For example, AC has high adsorption capacities for various organic pollutants, such as phenol, with a maximum adsorption capacity (q_max_) of 268.9 mg/g [14]. It is also effective in adsorbing radionuclides like uranium VI, with a q_max_ of 106 mg/g [21], and inorganic pollutants, including heavy metals such as lead (Pb^2^⁺), with a q_max_ of 249.3 mg/g [22].

The demand for AC is rising not only for new applications but also for its effectiveness in removing contaminants, particularly heavy metals [23]. Heavy metals are known to have adverse effects on the environment, and their concentrations are increasing [[24], [25], [26]], primarily due to activities such as metallurgy, dyeing operations, and pigment production [27]. A key characteristic of heavy metals is their non-biodegradability, allowing them to accumulate in organisms [28]. This accumulation facilitates their distribution across air, soil, and water systems [29,30].

Among heavy metals, chromium (Cr) is classified as one of the 16 most toxic metals [31,32], according to the World Health Organization (WHO). The maximum allowable concentration of chromium (VI) in drinking water is 0.05 mg/L [33], as it is the most toxic form of chromium, known to cause cancer in the digestive tract and lungs [34,35]. The adsorption of Cr(VI) is governed by several factors, including the characteristics of the raw materials and activating agents used in the preparation of activated carbon (AC), as well as the intrinsic physicochemical properties of the AC, such as surface chemistry and porosity. Furthermore, the pH of the solution plays a pivotal role in modulating the adsorption process. These factors collectively influence the speciation of chromium in the solution and the protonation of functional groups on the AC surface, thereby affecting the overall adsorption efficiency [36,37].

This study aims to explore the pyrolysis process of SCBs, a hazardous waste commonly found in urban environments. The thermodynamic parameters identified through thermo-kinetic analysis during pyrolysis will be used to optimize conditions to maximize the yield of desirable products, such as char, and enhance carbon sequestration. The char produced will be activated with potassium hydroxide (KOH) to increase its porosity and assess its capacity to adsorb chromium (VI). Our research provides a detailed overview of the pyrolysis and activation processes and their application in adding value to SCBs.

## 2 Materials and methods

### 2.1 Collection and preparation of non-smoked cigarettes and cigarette butts

In August 2021, smoked cigarette butts (SCBs) were collected from various commercial and social areas in Bogota. The collected SCBs were crushed using a cutting mill (homemade) and then sieved to obtain particles smaller than 250 μm in size (60 mesh). Additionally, non-smoked cigarette butts (CBs) were gathered from cigarettes that had not been smoked. For these, each filter was manually removed from each cigarette, and the same process of homogenization and size reduction was applied to achieve particles of the same size as those from the SCBs.

### 2.2 Preparation of activated carbon (AC)

The SCBs obtained in section 2.1 were subjected to pyrolysis at 350 °C to obtain char in a horizontal furnace (Thermolyne 21100). The pyrolysis process was conducted at a heating rate of 5 °C/min, with a nitrogen (Linde, 5.0) flow of 100 mL/min and a residence time of 120 min.

After the pyrolysis process, the resulting char was impregnated with a KOH solution for 24 h at a mass ratio of char to KOH (Merck, 85 %) of 1:5. The activation process was conducted under the following conditions: a heating rate of 5 °C/min, a maximum temperature of 800 °C, a nitrogen (Linde, 5.0) flow of 100 mL per minute, and a residence time of 120 min.

The activated carbon was washed with deionized water at temperatures above 60 °C until reaching a neutral pH. Finally, the sample was dried at 100 °C for 24 h.

### 2.3 Thermogravimetric experiment

Thermal stability and pyrolysis reactions were carried out with a thermal analyzer (Hitachi STA 7200 RV) with 100 mL/min nitrogen flow rate. About 8 mg of the sample was put in an alumina crucible and heated from 25 °C to 650 °C, with heating rates (β) of 5, 10, and 15 °C/min. Data from TGA analysis were used to determine the kinetic and thermodynamic parameters.

### 2.4 Kinetic analysis and thermodynamics

The pyrolysis kinetics of a residue are evaluated by the thermal degradation of solids into volatiles through a series of complex chemical reactions. As the temperature increases, the macromolecular structure of the residue undergoes progressive breakdown, leading to the formation of smaller components. This thermal degradation results in a continuous reduction in the mass of the solid phase [38].

The FWO, KAS, and Starink isoconversional methods are often used for the thermo-kinetic analysis in pyrolysis process. These models are based on the model-free approach, meaning they do not require an assumed reaction mechanism and can be applied without knowing the exact functional form of the conversion (g(α)).

The results from thermogravimetric analysis (TGA) were evaluated using isoconversional kinetic models, specifically the Kissinger-Akahira-Sunose (KAS), Ozawa-Flynn-Wall (OFW), and Starink (STK) methods. In kinetic analysis, various variables are used to describe the process. Alpha (α) represents the conversion of mass, while Beta (*β*) corresponds to the heating rate. The parameter *Ea* denotes the activation energy, and R is the ideal gas constant (8.314 kJ/(mol·K)). G refers to Gibbs free energy, and T is the temperature in Kelvin. Additionally, A is the frequency factor or pre-exponential factor, which is typically obtained using the Kissinger method (see Equation (1)) [38]. The Kissinger method does not yield an analytical solution, whereas the KAS, OFW, and STK models are derived using algebraic approximations.

[Eq. 1] $$g\left(\alpha \right)=\frac{A*E}{R*\beta }*P_{O_{2}}*\left[\frac{\exp\left(-x\right)}{x}-\int_{x}^{\infty }\frac{\exp\left(-x\right)}{x}dx\right]=\frac{A*E}{R*\beta }*P_{O_{2}}*p\left(x\right)$$

For the Kissinger-Akahira-Sunose (KAS) method, the activation energy is estimated with Equation (2) [38]:

[Eq. 2] $$\ln\frac{\beta }{T^{2}}=\ln\left(\frac{AR}{E_{a}G\left(\propto \right)}\right)-\frac{E_{a}}{RT}$$

The slope β/T^2^ is evaluated as a function of α, which represents the mass conversion relative to the temperature flux (β) [38]. For each value of α, the corresponding curves are generated using Equation (3):

[Eq. 3] $$\ln\frac{\beta }{T^{2}}\;\text{vs}\;\frac{1}{T}$$

The Ozawa-Flynn-Wall (OFW) method is represented by the following Equation (4) [38]:

[Eq. 4] $$\ln\left(\beta \right)=\ln\left[\frac{AE_{a}}{RG\left(\alpha \right)}\right]-5.331-1.052\left(\frac{E_{a}}{RT}\right)$$

The activation energy can be determined from the graph of ln(β) versus 1/T at each conversion. It is calculated from the slope of the resulting plot, as shown in Equation (5) [38]:

[Eq. 5] $$-1.052\left(\frac{E_{a}}{R}\right)$$

The Starink method (STK) [Eq. (6)] [38] offers a higher degree of precision compared to the OFW [Eq. (4)] and KAS [Eq. (2)] models. This improved precision is attributed to the algebraic methods employed, which enhance the accuracy of the equation. The STK model demonstrates similarities to a linear equation when compared to the KAS model. The STK model is represented by Equation (6):

[Eq. 6] $$\ln\left(\frac{\beta }{T^{1.92}}\right)=Const.-1.0008\left(\frac{E_{a}}{RT}\right)$$

To determine the activation energy, a plot of ln(β/T^1.92^) versus 1/T is generated, yielding a series of straight lines at different conversion levels. The slope of these lines, −1.0008(Ea/R), is then used to calculate the activation energy.

The Kissinger method is represented by Equation (7) [38]:

[Eq. 7] $$\ln\left(\frac{\beta }{T_{m}^{2}}\right)=-\frac{E_{\alpha }}{RT_{m}}+\ln\left(\frac{AR}{E_{a}}\right)$$

The Kissinger method is mainly used to estimate the frequency factor, using the activation energies obtained in each conversion using one of the different methods (See Equation (8)) [38]:

[Eq. 8] $$A=\frac{\beta E_{a}\;\exp\left(\frac{E_{a}}{RT_{m}}\right)}{RT_{m}^{2}}$$

In this context, β represents the heating rate, and Tm is defined as the temperature at the peak of the signal, as determined from the differential thermogravimetric (DTG) curve. The value obtained using Equation (8) is crucial for calculating the thermodynamic parameters.

Using the activation energy values, various thermodynamic parameters can be calculated, including the pre-exponential factor (A) from the Arrhenius equation, as well as enthalpy (ΔH), entropy (ΔS), and Gibbs free energy (ΔG). These parameters are determined through Equations [Eq. 9], [Eq. 10], [Eq. 11]) [38]:

[Eq. 9] $$\Delta H=E_{a}-RT$$

[Eq. 10] $$\Delta S=\frac{\Delta H-\Delta G}{T_{m}}$$

[Eq. 11] $$\Delta G=E_{a}+RT_{m}\;\ln\left(\frac{K_{b}T_{m}}{hA}\right)$$

Kb represents Boltzmann's constant ((1.38 × 10^−23^ J/K), and h represents Planck's constant (6.626 × 10^−34^ J/s). The activation energy required for the calculation of these thermodynamic parameters is determined using the KAS, OFW, and STK methods.

### 2.5 Determination of textural parameters

For the textural characterization of activated carbon from SCB (SCB-AC) and commercial activated carbon (CAC), N_2_ isotherms were performed at −192 °C in Autosorb iQ2 (Anton Paar). The samples were degassed at a temperature of 250 °C. Subsequently, the textural characteristics were determined: the BET surface area, micropore volume (Dubinin-Astakhov (DA) Method), Mesopore volume (Vol. Meso = Total Vol. - Vol. DA), and Total Volume (Volume adsorbed to 0.95 of P/P_0_).

### 2.6 Hexavalent chromium adsorption onto activated carbon

A stock solution (500 mg/L) of Cr(VI) was prepared by dissolving K_2_Cr_2_O_7_ (Merck, 99.0 %) in distilled water. The test solution of Cr(VI) used in each study was prepared by diluting the stock solution in the range of 5–300 ppm. Adsorption experiments were carried out by adding 50 mg activated carbon obtained from SCB samples into Erlenmeyer flasks containing 20 mL of Cr(VI) solution and a set temperature of 25 °C. The mixture was stirred using a magnetic stirrer at a constant stirring speed (150 rpm) for 24 h and filtered. Finally, for the quantification of the Cr (VI) content in each test, the pH was adjusted with solutions of 0.1 N HCl (Merck, 37 %) up to a value of 1 and then 200 μL of 1,5-diphenylcarbazide (Merck, ASC reagent) was added, and the absorbance of each of the samples was read at 540 nm in a UV–Vis Spectronic, Genesys 5. The values of Cr (VI) removed in each of the samples were calculated using a calibration curve of 20 mL of K_2_Cr_2_O_7_ as standard (0.1, 0.25, 0.5, 0.75, and 1 ppm, R^2^ = 0.99) and 200 μL of 1, 5-diphenylcarbazide at pH 1 [39]. All the adsorption experiments were performed in triplicate, and the average values were used for data analysis and modeling.

## 3 Results

### 3.1 Thermal decomposition analysis

**Fig. 1:**
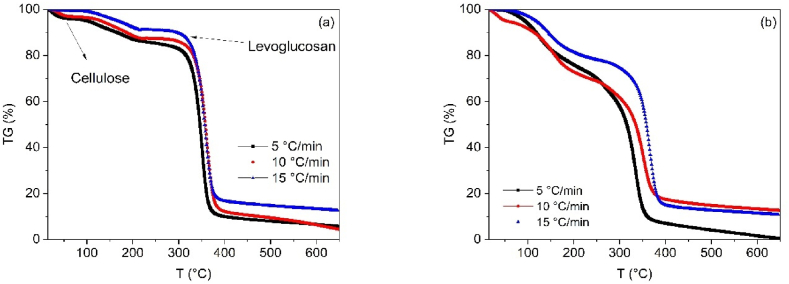
(a) Thermogravimetric curves for CB pyrolysis at 5, 10 and 15 °C/min. (b) Thermogravimetric curves for SCB pyrolysis at 5, 10 and 15 °C/min.

The thermal decomposition of SCBs and CB was evaluated using thermogravimetric analysis (TGA) under non-isothermal conditions (Fig. 1(a)–b). The TGA curves revealed notable variations in the behavior of the samples. Specifically, for the SCB samples, a distinct change in the decomposition trend was observed within the temperature range of 100–500 °C.

The thermal decomposition of cigarette butts (CB) follows a pattern similar to that of other biomass materials with a high cellulose content, such as date palm [40], sugar cane bagasse [41], and pine wood [42]. The results suggest that cigarette filters are composed of cellulose acetate [43], with their thermal degradation occurring within a temperature range of 300–400 °C. During this process, a product referred to as “active cellulose” is formed through the depolymerization of cellulose into levoglucosan, which eventually leads to the production of volatile species (g) and char (s). Additionally, it is observed that at temperatures below 200 °C, a dehydration process occurs in which water molecules bound between cellulose chains are eliminated.

A distinct peak is observed in the differential thermogravimetric (DTG) curve of the CB at 340 °C, corresponding to the temperature at which the maximum conversion rate occurs. The CB remain stable up to 300 °C, after which the degradation of the primary component, cellulose, begins. In this process, the mass loss associated with moisture content ranges between 1 and 4%. The degradation of cellulose contributes significantly to the overall mass loss, accounting for approximately 75–76 %. At a temperature of 600 °C, the yield is approximately 9.59 %.

In contrast, the thermal decomposition of SCBs is influenced by the additional organic compounds present, which are released during the combustion of the tobacco in the cigarette. These products are retained within the filter, causing differences in the degradation rates observed in the DTG curves. Despite the shared primary component, cellulose acetate, the presence of these additional compounds in SCBs alters the thermal behavior, as evidenced by the differences in the TGA and DTG results.

**Fig. 2:**
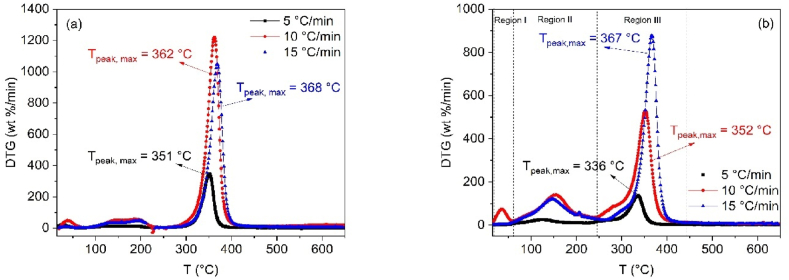
(a) DTG curves for CB pyrolysis at 5, 10 and 15 °C/min and (b) DTG curves for SCB pyrolysis at 5, 10 and 15 °C/min.

To analyze the changes in thermal behavior, differential thermogravimetric (DTG) curves for both CB and SCB samples were determined. As shown in Fig. 2(a), the thermal decomposition of CB occurs within a temperature range of 245–440 °C, with a significant mass loss of 75–76 % in region III, primarily attributed to the degradation of cellulose. The DTG curves reveal that the degradation processes are influenced by the heating rate. Specifically, the highest mass loss percentages and the greatest reactivity of CB are observed at heating rates of 10 and 15 °C/min. In contrast, at a heating rate of 5 °C/min, the reactivity is lower, likely due to the reduced activation energy supplied at the slower rate.

Conversely, SCBs exhibit a different thermal behavior, as shown in Fig. 2(b). At all three heating rates (5, 10, and 15 °C/min), the DTG curves for SCBs reveal additional processes compared to CB. For heating rates of 10 and 15 °C/min, three distinct regions are observed. The first region, between 25 and 60 °C, corresponds to the evaporation of absorbed volatile compounds, such as 1,3-butadiene, formaldehyde, and isoprene. The second region, spanning 60–250 °C, involves the vaporization of organic compounds with higher boiling points, such as benzene, benzo[a]pyrene, and acrylonitrile [44]. The third region, between 250 and 400 °C, corresponds to the degradation of cellulose acetate (the primary component of SCBs), resulting in the formation of a solid residue (carbonized material) [45].

When comparing the DTG curves of CB and SCB, it is evident that the SCBs show variations in peak behavior. These differences are attributed to the presence of additional chemical compounds retained within the cigarette filter, which are released during the combustion of tobacco. In contrast, CB mainly consists of cellulose acetate, with no additional compounds from tobacco combustion.

### 3.2 Effect of heating rate

Fig. 2(a) and (b) illustrate the effect of heating rate on the thermal decomposition of CB and SCB. While the DTG curves for both samples exhibit similar shapes at each heating rate, the key difference lies in the SCB sample, where additional chemical substances, derived from the smoking process, are present prior to pyrolysis, as discussed in section 3.1. For CB, the peak temperatures shift from 351 °C to 368 °C as the heating rate increases, corresponding to the degradation of cellulose acetate. In contrast, the peak temperatures for SCB are observed at 336 °C, 352 °C, and 367 °C. This shift indicates that the heat transfer between the environment and the system is not uniform, leading to a decrease in the degradation rate and a displacement of the curves with increasing heating rate.

**Fig. 3:**
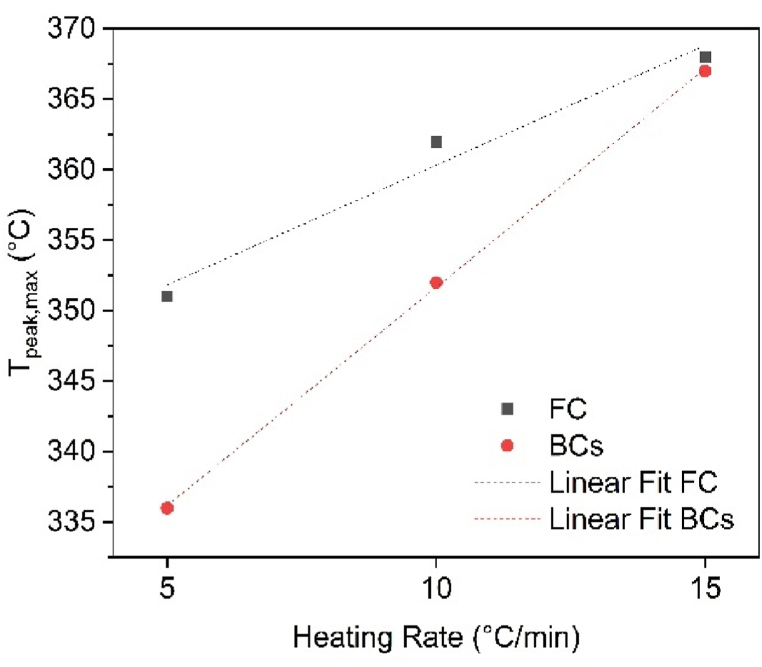
Effect of heating speed on Tpeak, max of CB and SCB.

Furthermore, a comparison of the two samples reveals a reduction in the maximum degradation temperatures (T_max_) for SCBs, which can be attributed to the presence of additional compounds absorbed during tobacco combustion. These compounds enhance heat transfer, thereby lowering T_max_. However, at a heating rate of 15 °C/min, the T_max_ for both CB and SCB is similar. This suggests that at this heating rate, the thermal transport facilitated by the absorbed molecules in SCBs is inhibited by rapid vaporization processes, resulting in the degradation of cellulose acetate at comparable temperatures for both samples (see Fig. 3).

### 3.3 Kinetic analysis

Pyrolysis of cigarette butts involves multiple components, such as cellulose acetate and organic material (tobacco remnants). While pyrolysis is inherently a multi-step process, the char formation step is often treated as a single-step reaction in kinetic studies. This simplification is valid because the formation of char, primarily from cellulose acetate, can be considered as the dominant solid-phase process during pyrolysis.

**Fig. 4:**
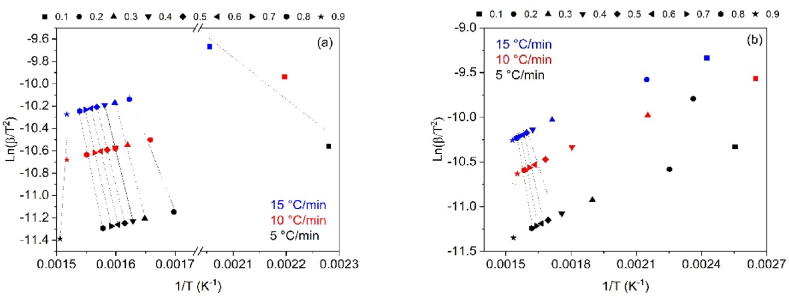
(a) KAS model for the determination of CB Activation Energy and (b) KAS model for SCB activation energy determination at 5, 10 and 15 °C/min.

**Fig. 5:**
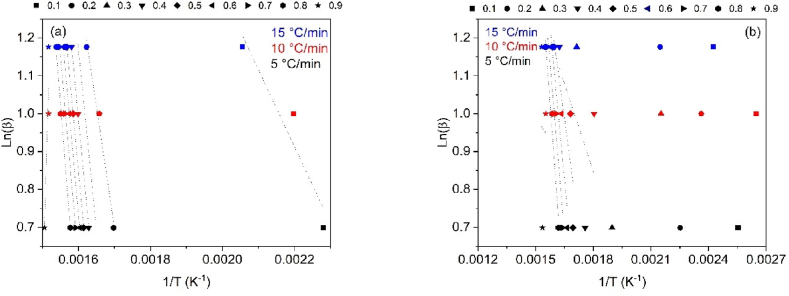
(a) OFW model for the determination of CB activation energy and (b) OFW Model for SCB activation energy determination at 5, 10 and 15 °C/min.

**Fig. 6:**
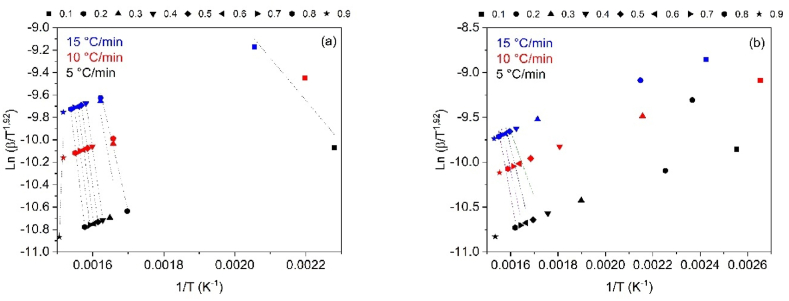
(a) STK model for the determination of CB activation energy and (b) STK model for the determination of SCB activation energy at 5, 10 and 15 °C/min.

To determine the thermal degradation mechanism of CB and SCB samples, the linear kinetic isoconversion models Kissinger-Akahira-Sunone (KAS), Osawa-Flynn-Wall (OFW), and Starink (STK) were employed. Fig. 4(a–b) present the results obtained using the KAS model for CB and SCB, while Fig. 5(a–b) demonstrate the application of the OFW model. The results related to the STK model are shown in Fig. 6(a–b). Activation energy (Ea) values were determined using Equations [Eq. 4], [Eq. 5]), with Ea calculated for each conversion between 10 % and 90 %. The average activation energies for CB were found to be 216.77 kJ/mol (KAS), 176.31 kJ/mol (OFW), and 217.08 kJ/mol (STK), while for SCB, the average Ea values were 46.73 kJ/mol (KAS), 53.32 kJ/mol (OFW), and 46.10 kJ/mol (STK).

The results reveal that for CB, the Ea values from the KAS and STK models are similar, while a significant deviation of 23.45 kJ/mol is observed in the Ea value from the OFW model. In contrast, the Ea values for SCB show a smaller deviation of 4.00 kJ/mol across the three models. Fig. 6(a) demonstrates that for CB, the three models exhibit similar behavior, with Ea increasing with conversion from 0.1 to 0.8, corresponding to temperatures between 165 and 360 °C. This increase is associated with the energy required for the thermal decomposition of cellulose acetate, with an Ea value of 216.77 kJ/mol, which is consistent with previously reported values ranging from 135 to 223 kJ/mol [46].

**Fig. 7:**
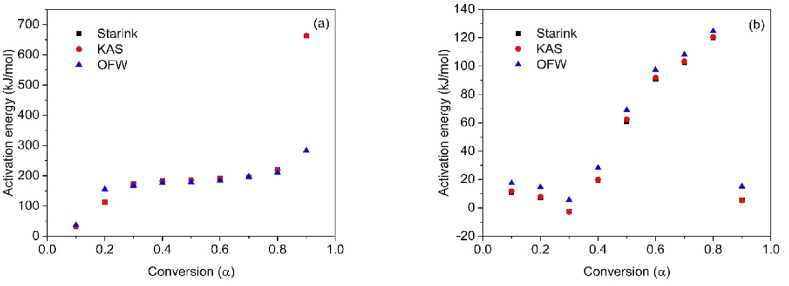
(a) Change in activation energy with respect to conversion without using STK, KAS and OFW CB methods and (b) Change in activation energy with respect to the conversion of SCB methods STK, KAS and OFW.

For SCB, a different trend is observed: Ea decreases in the conversion interval of 0.1–0.3, while in the 0.4–0.8 range, Ea increases. Notably, at a conversion of 0.9, Ea decreases by approximately half of the value obtained at 0.1 for the KAS and STK models. In the OFW model, Ea at α = 0.9 is similar to that at α = 0.1. The average Ea for SCB is 48.72 kJ/mol, which aligns with the values reported by other authors, ranging from 36.56 to 38.47 kJ/mol [30]. Fig. 7(a–b) demonstrate the variations in Ea in relation to conversion for CB and SCB. These figures highlight the distinct thermal patterns observed in each type. In particular, Fig. 7(b) shows how the generation of additional organic compounds, such as nicotine and tar, during tobacco combustion influences the thermal behavior of SCB.

The linear models used in this study produced correlation coefficients ranging from 0.8644 to 0.9999 for the CB samples. The maximum mean absolute percentage error was found to be 6.7 % for the OFW model at α = 0.9, indicating a model accuracy of at least 93.3 %. For the other models, the accuracy is even higher, with root mean square deviations (RMSD) ranging from 0.02 to 0.2, indicating a minimal spread of data. The KAS model provided the best balance between model complexity and goodness of fit, as assessed by the corrected Akaike Information Criterion (AICc), which ranged from 60 to 87.

For specific conversions, such as KAS at α = 0.2, low correlation coefficients (R^2^ = 0.0037) were observed, likely due to the multiple processes occurring from the adsorption of various organic compounds in the filter after smoking. The maximum mean absolute percentage error for SCBs was found to be 20.8 % for the OFW model at α = 0.9, corresponding to an accuracy of at least 79.2 %. In contrast, the maximum mean absolute percentage errors for the KAS and STK models did not exceed 4.5 %. The KAS model again showed the best compensation between model complexity and goodness of fit, with AICc values ranging from 46 to 77.

### 3.4 Thermodynamic analysis

**Fig. 8:**
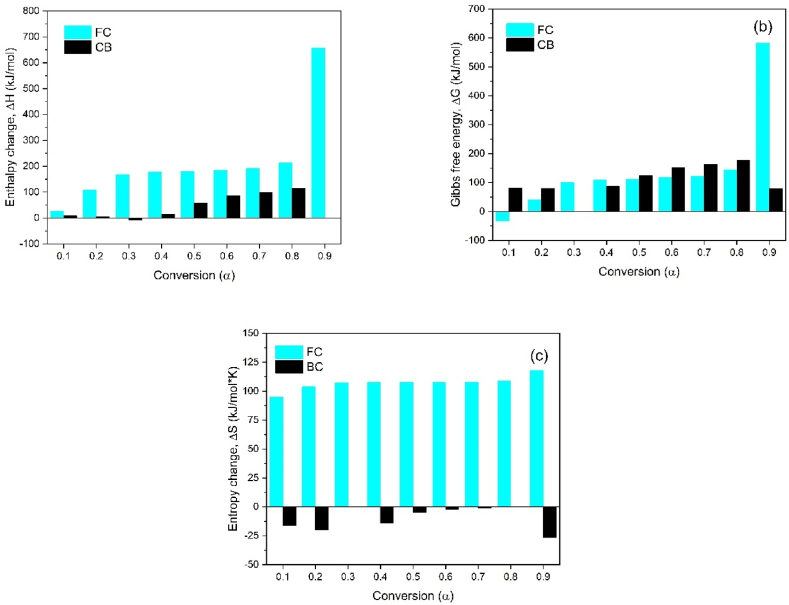
(a) Change in enthalpy as a function of conversion, (b) Change in free energy as a function of conversion and (c) Change in entropy as a function of conversion.

The thermodynamic parameters (ΔH, ΔG, ΔS) and the pre-exponential factor (A) for the pyrolysis of CB and SCB were determined using the KAS model, as shown in Fig. 8(a)–(c). The enthalpy change (ΔH) values for CB exhibit positive values across the conversion range of 0.1–0.9, indicating the endothermic nature of the pyrolysis process. In contrast, for SCB, the enthalpy values are approximately 20 % lower than those of CB. This reduction can be attributed to the presence of compounds absorbed during the tobacco combustion process, which require less energy to undergo phase changes and form pyrolysis products. Notably, at a conversion of 0.3, a negative enthalpy value is observed, signifying an exothermic process associated with the decomposition of low molecular weight compounds.

Regarding the Gibbs free energy (ΔG), both CB and SCB exhibit positive values, indicating that the pyrolysis reactions are non-spontaneous and require an input of energy. On average, the ΔG for CB is 143.85 kJ/mol, while for SCB, the average ΔG is 117.59 kJ/mol. These values are consistent with those reported by Alves et al. [38], who found Gibbs free energy changes (ΔG) in the range of 122.63–175.43 kJ/mol for the pyrolysis of smoked cigarette butts.

The entropy (ΔS) values for CB are consistently positive across the entire conversion range, which can be attributed to the dominant role of cellulose acetate pyrolysis products in the decomposition process. In contrast, for SCB, negative entropy values are observed throughout the entire conversion range. This is indicative of the ordering effect in the char produced, suggesting a more organized structure as the pyrolysis process progresses. These negative entropy values imply that, despite the occurrence of multiple reactions, including devolatilization and decomposition, a thermal equilibrium is reached, resulting in the formation of a thermally stable char. Such behavior is consistent with the characteristics observed in biomass pyrolysis processes, where complex reactions lead to the stabilization of solid carbonaceous residues [47].

### 3.5 Textural analysis

The yield obtained from thermogravimetric analysis (TGA) was 29.64 %, a result that was corroborated by the yield observed during the carbonization process in a horizontal furnace. During the activation process, the char obtained at a heating rate of 5 °C/min was selected for further activation, as it exhibited a higher char yield compared to those processed at heating rates of 10 and 15 °C/min. The lower heating rate favors the production of char by minimizing the release of volatile matter, thus increasing the proportion of carbon retained in the char form. This higher char content serves as a better precursor for the activation process, enhancing the development of porosity in the activated carbon (AC). The activation process yielded a char recovery of 53.55 %, a value consistent with those reported in similar studies [48].

**Fig. 9:**
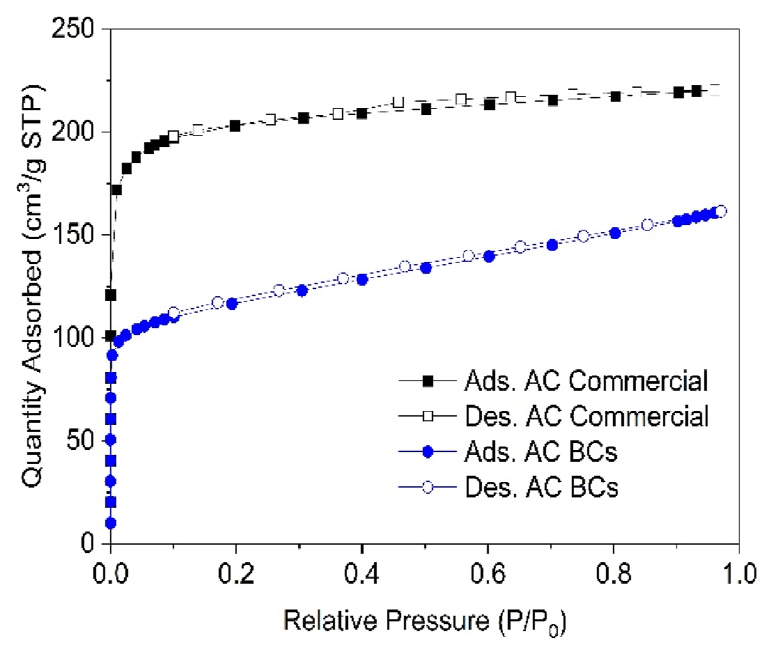
Nitrogen isotherms at −196 °C for CAC and SCB-AC.

The textural properties of the activated carbon derived from smoked cigarette butts (SCB-AC) were evaluated through nitrogen adsorption at −196 °C (see Fig. 9). To facilitate comparison, commercial activated carbon (CAC) derived from coconut shell was also analyzed, as it is commonly used in various adsorption processes involving aqueous solutions [49]. The adsorption isotherms for both SCB-AC and CAC were classified as Type I (b) according to the IUPAC classification [50], indicating that both materials are porous, with pore size distributions falling within the range of narrow micropores and mesopores (below 2.5 nm). The specific surface area, micropore volume (using the Dubinin-Astakhov model), and total volume were determined at a P/P_0_ value of 0.95, and the results are summarized in Table 1.

**Table 1:** Commercial AC and SCB AC textural parameters.

Textural Parameters	Commercial AC	AC of SCB
BET surface area (m^2^/g)	783	434
Micropore volume (cm^3^/g)	0.33	0.17
Mesopore volume (cm^3^/g)	0.01	0.08
Total Pore volume (cm^3^/g)	0.34	0.25

Notably, the SCB-AC exhibited a distinct change in slope between P/P_0_ values of 0.1 and 0.9, which is indicative of its interconnected pore structure. In contrast, the CAC showed no significant change in slope, suggesting limited pore connectivity or interconnectivity. The SCB-AC had a surface area of 434 m^2^/g, while the CAC had a surface area of 783 m^2^/g (see Table 1). The corresponding micropore volumes were similar for both materials: 0.33 cm³/g for SCB-AC and 0.33 cm³/g for CAC, reflecting a similar volume distribution of smaller pores. Additionally, the C parameter in the BET equation indicates a strong interaction between the adsorbate and the adsorbent in both cases.

Interestingly, the SCB-AC isotherm demonstrated a slope within the P/P_0_ range of 0.1–0.8, suggesting the development of mesoporosity due to the effect of the potassium hydroxide (KOH) activation agent [51]. The SCB-AC exhibited a higher proportion of mesoporosity (32 %) compared to the CAC (3 %), which aligns with the activation mechanism using KOH. Under higher activation energy conditions, greater porosity is generated as a result of the gasification processes that occur during the material activation [52].

**Fig. 10:**
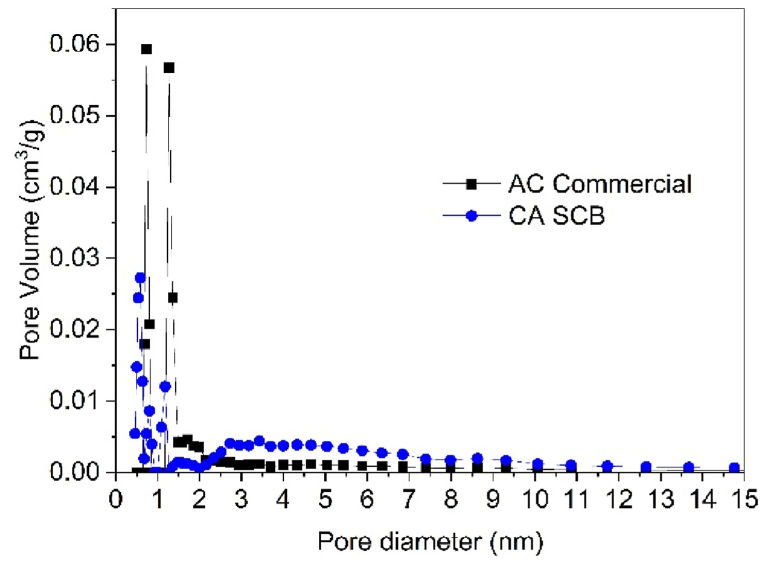
Pore Size Distribution for the CAC and SCB-AC samples.

Fig. 10 presents the pore size distribution obtained through the application of Density Functional Theory (DFT) analysis. The micropore distribution of the commercial activated carbon (CAC) sample shows peaks at 0.7 and 1.2 nm, whereas the smoked cigarette butt-derived activated carbon (SCB-AC) exhibits peaks at 0.6, 0.8, and 1.7 nm. In terms of mesoporosity, the CAC sample demonstrates a more homogeneous distribution, with pore sizes ranging from 2 to 6 nm. In contrast, the SCB-AC sample displays a broader mesopore distribution, extending from 2 to 8 nm.

### 3.6 Chromium(VI) adsorption from aqueous solution

#### 3.6.1 Cr(VI) adsorption kinetics

**Fig. 11:**
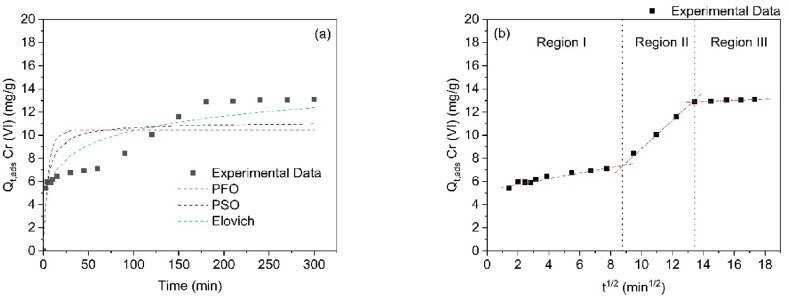
(a) Cr(VI) adsorption kinetics on SCB-AC (b) Morris and Weber intraparticle diffusion model.

The kinetics of Cr(VI) adsorption onto SCB-AC were investigated to assess its efficiency, employing various non-linear kinetic models, including the pseudo-first-order (PFO), pseudo-second-order (PSO), and Elovich models, as shown in Fig. 11(a). The PFO model assumes that the adsorption process is controlled by the diffusion of the adsorbate, forming a monolayer via boundary diffusion [53]. In contrast, the PSO model assumes that adsorption is governed by chemical interactions, such as the transfer or sharing of electrons, between the adsorbate and adsorbent [54]. The Elovich model, on the other hand, postulates that the adsorption sites on the material are heterogeneous, leading to varying activation energies [55].

The results, as presented in Table 2, indicate that the kinetic data are best described by the Elovich model, with a correlation coefficient (R^2^) of 0.8832, followed by the PSO (R^2^ = 0.7013) and PFO (R^2^ = 0.5621) models. As illustrated in Fig. 11(a), the adsorption process can be divided into distinct stages. The first stage, occurring within the initial 60 min, is associated with the mass transfer of Cr(VI) from the solution, primarily governed by external diffusion. This is followed by a second stage, between 60 and 170 min, where the adsorbate diffuses into the internal pores of the SCB-AC, representing internal diffusion. Finally, equilibrium is reached after 170 min, with the remaining interactions occurring between the adsorbate and the internal surface of the SCB-AC. This observed behavior aligns with findings reported in the removal of hexavalent chromium using polyethyleneimine-impregnated activated carbon [56].

**Table 2:** Kinetic parameters of Cr (VI) adsorption on AC obtained from SCB.

Model	Non-Linear Model			Cr (VI)
Pseudo First Order (PFO)	$q_{t}=q_{e}\left(1-e^{-k1∙t}\right)$		q_1_ (mg g^−1^)	10.45
			K_1_ (min^−1^)	0.187
			R_1_^2^	0.5621
Pseudo Second Order (PSO)	$q_{t}=\frac{q_{2}^{2}k_{2}t}{q_{e}k_{2}t+1}$		q_2_ (mg g^−1^)	11.12
			K_2_ (g mg^−1^•min^−1^)	0.02
			R_2_^2^	0.7013
Elovich	$q=\left(\frac{1}{\beta }\right)\mathrm{Ln}\left(1+\alpha \beta t\right)$		α (mg g^−1^)	5.76
			β (g mg^−1^•min^−1^)	0.55
			R_E_^2^	0.8832
Weber and Morris	$q=k_{in}t^{0.5}+C$	Region I	K_in,1_ (mg g^−1^ min^−1/2^)	0.24
			C (mg g^−1^)	5.33
			R^2^	0.9362
		Region II	K_in,2_ (mg g^−1^ min^−1/2^)	1.14
			C (mg g^−1^)	–
			R^2^	0.9996
		Region III	K_in,3_ (mg g^−1^ min^−1/2^)	0.05
			C (mg g^−1^)	12.17
			R^2^	0.9219

The kinetic data obtained from the Elovich model suggest that the adsorption process follows the intra-particle diffusion model. As shown in Fig. 11(b), the adsorption process is divided into three distinct regions, each characterized by different diffusion rates. The diffusion rates corresponding to each region were determined, with region II exhibiting the highest contribution (1.14), followed by region I (0.24) and region III (0.05), as summarized in Table 2. These results indicate that the external diffusion of Cr(VI) to the adsorbent surface occurs at a slower rate. Once the external diffusion is overcome, the rate of diffusion increases by a factor of 4.75 in region II, as the process transitions into the porosity of the adsorbent, which corresponds to physical adsorption. Finally, in region III, the adsorption reaches equilibrium, primarily through interactions with surface groups (chemical adsorption), completing the process within approximately 200 min [57].

#### 3.6.2 Cr(VI) adsorption isotherms

**Fig. 12:**
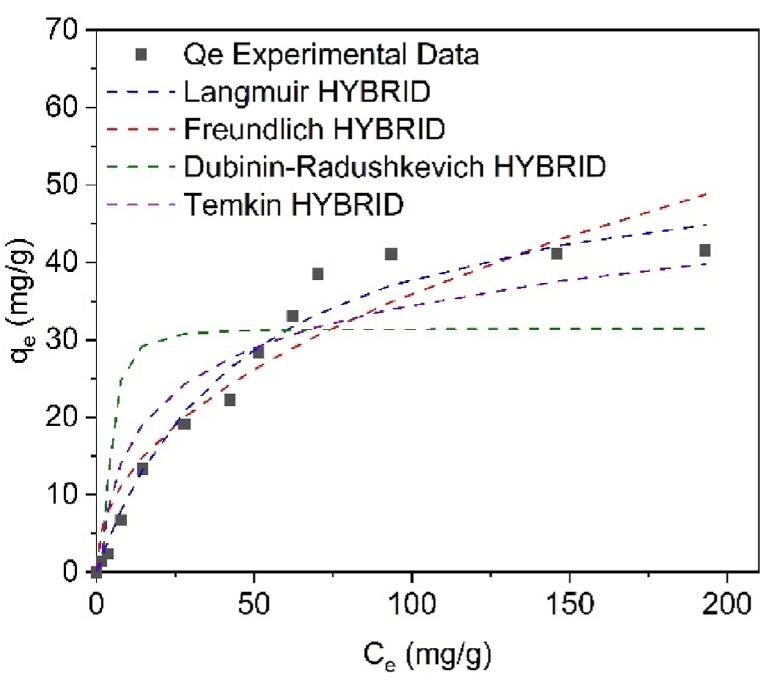
Cr(VI) adsorption isotherms on SCB-AC. Conditions: T = 20 °C, pH = 1.0, Equilibrium Time 24 h.

Fig. 12 presents the Cr(VI) adsorption isotherms, which were fitted to the Langmuir, Freundlich, Dubinin-Raduskevich, and Temkin models. The theoretical and experimental data were evaluated using various error functions, including SSE, MPSD, EABS, ARE, R^2^, and HYBRID. The results indicate that the best fit to the experimental data was obtained using the HYBRID model, which yielded the minimum error [58]. Based on this, it was determined that the Langmuir model provided the best representation of the data, with an R^2^ value of 0.9719 (see Table 3). The Langmuir model assumes that (I) the adsorption process forms a homogeneous monolayer, (II) there is no interaction between adjacent adsorbed ions, and (III) the adsorption sites are limited.

**Table 3:** Parameters obtained from the adjustments of Cr(VI) adsorption models on AC obtained from SCB.

Model	Parameters	Cr (VI)
Langmuir	q_max_ (mg g^−1^)	55.8
	K_L_ (L/mg)	0.02
	R^2^	0.9719
Freundlich	n	0.46
	K_F_ (L/mg)	4.30
	R^2^	0.8876
Dubinin-Radushkevich	β (mol^2^•kJ^−2^)	3.7 × 10^−5^
	E_L_ (kJ/mol)	34.29
	R^2^	0.7388
Temkin	K_T_ (L/mol)	0.74
	b (J/mol)	301.7
	R^2^	0.9336

Using the Langmuir constant (KL) presented in Table 3, the separation factor (RL) was calculated to evaluate the characteristics of the adsorption process. The RL values obtained for Cr(VI) concentrations of 5, 50, 100, 150, and 300 ppm were 0.9, 0.5, 0.3, 0.2, and 0.1 (nondimensional), respectively. According to these values, it can be inferred that the Cr(VI) adsorption process on SCB-AC is favorable, as indicated by RL values between 0 and 1, which correspond to favorable adsorption conditions.

A comparison is made between various adsorbates that have been researched over the last five years, using SCB as a precursor to produce activated carbon (AC). It is important to highlight that the material obtained demonstrates significant versatility in removing a wide range of contaminants, both organic and inorganic. The adsorption capacity of the SCB-derived AC is influenced by the preparation process, which results in varying textural characteristics and surface chemistry. These differences lead to distinct adsorption mechanisms, which can be selective depending on the specific application (see Table 4).

**Table 4:** Comparative table for adsorption of organic and inorganic pollutants on AC obtained from BCs.

Adsorbate	Method	Q_max_ Langmuir (mg/g)
Pb^2+^, Cr^3+^, Ni^2+^, Cd^2+^	Carbonization Hydrothermal, Activation with KOH	109.8–209.6 Pb^2+^ [22]
Roxarsone	Carbonization Hydrothermal, Pyrolysis	697 [59]
Chloramphenicol	Carbonization Hydrothermal, Activation with K_2_CO_3_	450 [60]
Phenol	Pyrolysis, Activation with CO_2_	272 [14]
Phenacetin	(Tin chloride pentahydrate + KOH), Pyrolysis	156.4 [61]
Bisphenol A	Carbonization Hydrothermal, Pyrolysis	364.2 [62]
Cr^6+^	Pyrolysis, Activation with KOH	55.8 (In this research)

Finally, a comparison is made between various carbonaceous materials used in the removal of Cr (VI) over the past five years (see Table 5). It is observed that the adsorption capacities of these materials depend on both the precursor used and the method employed in the preparation of the activated carbon (AC). The results presented indicate that many of the materials utilized are derived from waste residues commonly found in specific regions, offering a high availability of precursors. However, cigarette butts (SCBs) represent a particularly prevalent waste stream, which continues to pose an environmental challenge. By utilizing SCBs as a precursor, added value is provided to this waste, enabling its use in the removal of Cr (VI) — a substance commonly found in alloys, pigments, and wood preservatives.

**Table 5:** Comparative table of Cr (VI) adsorption on different AC obtained by different precursors.

Precursor	Q_max_ Langmuir (mg/g), Cr^6+^
Polyethyleneimine (PEI)/Activated carbon from softwood pulp	1.58 [63]
Bermuda Grass	403.23 [64]
Petroleoum coke Feedstock	22.4 [65]
Soybean Straw	294.12 [66]
Tires Waste	142.85 [66]
Spent Coffee grounds	187.6 [67]
Somoken Butts Cigarettes	55.8 (In this research)

## 4 Conclusion

The thermodynamic parameters obtained applying FWO, KAS, and Starink methods are critical for optimizing pyrolysis conditions to maximize desired products yields and play a crucial role in designing large-scale pyrolysis reactors.

In conclusion, the production of activated carbon from the pyrolysis of smoked cigarette butts (SCBs) followed by KOH activation resulted in a highly porous material with a specific surface area of 434 m^2^/g. This activated carbon exhibited promising adsorption capabilities, particularly in the removal of Cr (VI), achieving a notable maximum adsorption capacity (Q_max_) of 55.8 mg/g, as determined by the Langmuir model.

The kinetic study identified a three-step adsorption mechanism involving external and internal diffusion, followed by interactions between the Cr (VI) adsorbate and the active sites within the AC structure. This mechanism was supported by the high correlation (R^2^ = 0.9719) between the Langmuir model and the adsorption isotherm, further validating the proposed adsorption process.

Additionally, the textural parameters emphasized the crucial role of microporosity and mesoporosity in facilitating internal diffusion processes. These pore sizes were instrumental in enabling the movement and interaction of the adsorbate within the activated carbon matrix, underlining the importance of pore structure in optimizing adsorption performance.

## CRediT authorship contribution statement

**Carlos Felipe Herrera-Puerta:** Investigation, Data curation, Conceptualization. **Santiago Suspes-García:** Formal analysis, Data curation, Conceptualization. **Diana M. Galindres-Jimenez:** Methodology, Formal analysis, Data curation, Conceptualization. **Diego Cifuentes-Galindres:** Supervision, Investigation, Formal analysis, Data curation, Conceptualization. **Luz Elena Tinoco:** Writing – original draft, Methodology, Formal analysis, Data curation, Conceptualization. **Juan Carlos Moreno Piraján:** Writing – review & editing, Writing – original draft, Supervision, Methodology, Investigation, Funding acquisition, Formal analysis, Data curation, Conceptualization. **Liliana Giraldo Gutierrez:** Writing – review & editing, Writing – original draft, Methodology, Investigation, Formal analysis, Conceptualization. **Yesid Murillo-Acevedo:** Writing – review & editing, Writing – original draft, Validation, Supervision, Methodology, Investigation, Formal analysis, Data curation, Conceptualization.

## Data availability statement

Data will be made available on request.

## Ethics declaration

Review and/or approval by an ethics committee as well as informed consent was not required for this study because this article did not involve any direct experimentation/studies on living beings.

## Declaration of competing interest

“We declare no conflicts of interest in our experimentation."
